# Supplementary material for: Scalable Microbial Strain Inference in Metagenomic Data Using StrainFacts
Source: Front Bioinform. 2022 May 16;2:867386. doi: 10.3389/fbinf.2022.867386 (PMC9580935; doi:10.3389/fbinf.2022.867386)
Supplement: Supplementary file 1 [file DataSheet1.docx]

Scalable microbial strain inference in metagenomic data using StrainFacts

Byron J. Smith ^A,B^ (ORCID: 0000-0002-0182-404X)

Xiangpeng Li ^C^

Zhou Jason Shi ^A,D^

Adam Abate ^C,D^ (ORCID: 0000-0001-9614-4831)

Katherine S. Pollard ^A,B,D,*^ (ORCID: 0000-0002-9870-6196)

^A^ The Gladstone Institute of Data Science and Biotechnology, San Francisco, CA
^B^ Department of Epidemiology and Biostatistics, University of California, San Francisco, CA
^C^ Department of Bioengineering and Therapeutic Sciences, University of California, San Francisco, CA
^D^ Chan-Zuckerberg Biohub, San Francisco, CA

* Corresponding author:
[katherine.pollard@gladstone.ucsf.edu](mailto:katherine.pollard@gladstone.ucsf.edu)

# Supplementary Materials

## Supplementary Methods


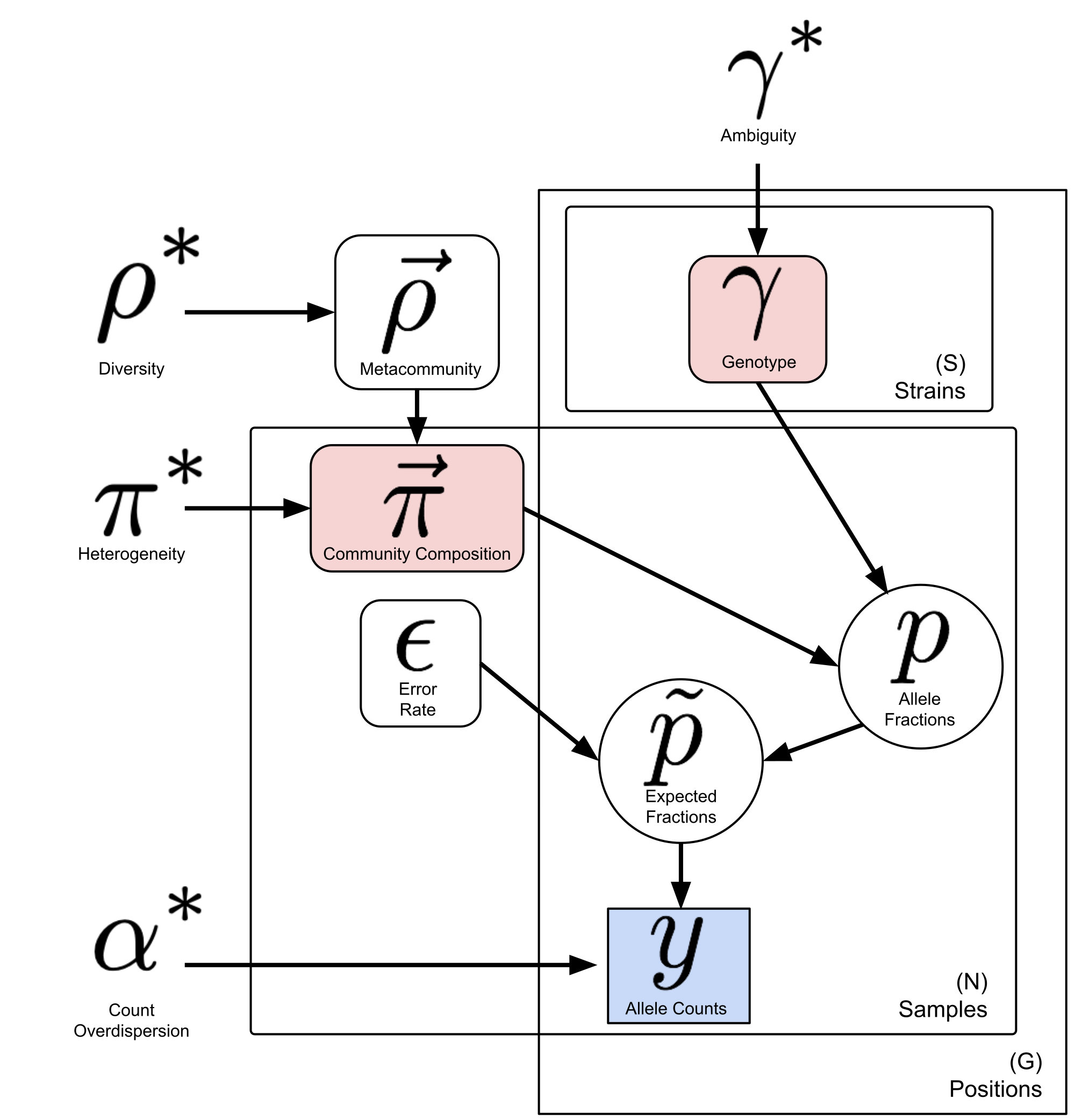


**Figure S1:** Graphical representation of the StrainFacts model including hyperparameters. Symbols include observed data (blue box), deterministic terms (circles), key parameters being estimated (red boxes), and key hyperparameters (unenclosed). Plates behind terms indicate the dimensionality and indexing of the variables and arrows connect the terms that directly depend on one another.

### The shifted, scaled Dirichlet distribution

The k-dimensional SSD is a 2k + 1 parameter family which includes the Dirichlet distribution as a special case, and is defined by Aitchenson “perturbation” ($\oplus$) and “powering” ($\odot$) operations ([Aitchison, 1986](#ref-Aitchison1986)) applied to a Dirichlet-distributed random variable. Given a random variable $\mathbf{X}\sim Dirichlet\left( \boldsymbol{\alpha} \right), \boldsymbol{\alpha}\in\mathbb{R}^{K}, \mathbf{X}\in\mathcal{S}^{D}$ if $\mathbf{Y}=\mathbf{p}\oplus\left( a\odot\mathbf{X} \right), a\in\mathbb{R}_{+}, \mathbf{p}\in\mathcal{S}^{K}, \mathbf{Y}\in\mathcal{S}^{K}$ then $\mathbf{Y}\sim SSD\left( \boldsymbol{\alpha},\mathbf{p},a \right)$.

In this work, we limit our use of this distribution to $\boldsymbol{\alpha}=\mathbf{1}$, i.e. $\mathbf{X}$ distributed uniformly on the K-simplex before powering and perturbation. For values of $a>1$, the probability mass shifts towards the edges of the simplex, and we use this property in order to induce sparsity in our estimates.

### Parameter initialization and optimization

In select initial values of $\boldsymbol{\Pi}$ and $\boldsymbol{\Gamma}$ using an NMF based approach. First, we transform metagenotypes from counts to an $N\times G\times2$ matrix of allele frequencies, and stack this into an $N\times2G$ matrix, $\mathbf{P'}$, with columns of reference alleles followed by columns of alternative alleles. We then use canonical NMF—implemented in the scikit-learn library ([Pedregosa et al., 2011](#ref-Pedregosa2012))—to factorize this data matrix into $\boldsymbol{\Pi'}$ and $\boldsymbol{\Gamma'}$, where $\mathbf{P'}\approx\boldsymbol{\Pi'}\times\boldsymbol{\Gamma'}$ with a shared, inner dimension of size $S$. After reversing the stacking of alleles, we get back a matrix $\boldsymbol{\Gamma''}\in\mathbb{R}_{+}^{S\times G\times2}$. Since $\boldsymbol{\Pi'}$ and $\boldsymbol{\Gamma''}$ likely do not conform to the constraints of strain deconvolution, we transform them into initial values as follows: $\boldsymbol{\Gamma}_{\mathrm{init}}\mathcal{=C}\left( 1/\vec{c}*\boldsymbol{\Gamma''} \right)$ and $\boldsymbol{\Pi}_{\mathrm{init}}\mathcal{=C}\left( \vec{c}*\boldsymbol{\Pi'} \right)$ where $c_{s}=\frac{1}{G}\sum_{g} \left( \gamma_{sg0}+\gamma_{sg1} \right)$ , $*$ denotes element-wise multiplication, and $\mathcal{C}\left( \cdot\right)$ is normalization over the last dimension to the standard simplex (i.e. summing-to-one).

Model parameters are transformed to the unconstrained space using Pyro’s built-in defaults. Parameters other than $\boldsymbol{\Pi}$ and $\boldsymbol{\Gamma}$ are initialized randomly to a point on the interval $\left( -2,2 \right)$ in the transformed space. We then apply the Adamax algorithm for stochastic gradient descent using an initial learning rate lr_init. To increase the probability of finding a global maximum, we take a prior annealing approach ([Neuwald and Liu, 2004](#ref-Neuwald2004)): for an initial n_wait number of steps of the optimization routine, the hyperparameters $\gamma^{*}$ and $\rho^{*}$ are set to initial values with less stringent regularization and are then exponentially relaxed to their final values during the next n_anneal steps. After this annealing period, we continue taking gradient steps until the value of the loss function has not improved for 100 steps, at which point we halve the learning rate. Optimization is stopped when the learning rate falls below a minimum threshold, lr_min.

### Fitting full length genotypes

Because many metagenotypes had more than 5,000 SNP sites, we use a refitting approach to get full length strain genotypes. This is accomplished by conditioning our model on both the observed data and the previously estimated $\boldsymbol{\Pi}$. In addition, we update the value of two of the hyperparameters; we set $\gamma^{*}=1.0$, and $\alpha^{*}=200$. After refitting the other parameters, this results in a new estimate of $\boldsymbol{\Gamma}$. Since SNPs are statistically separable when $\boldsymbol{\Pi}$ is conditioned out, this allows us to iteratively refit arbitrary subsets of SNPs before recombining them into a full length genotype matrix.

### Single-cell genomic sequencing

#### Cell isolation from stool samples

Bacterial cells were isolated from fecal samples according to previously published protocol ([Hevia et al., 2015](#ref-Hevia2015)) with modifications. Briefly, 0.2-0.5 g of fecal samples were homogenized in 10 mL of PBS buffer by vertexing. After filtering with a 50 µm cell strainer (Corning, 431752) to remove most of the fecal particles, the flow through suspension was loaded on top of 3.5 mL of 80% Nycodenz® (Cosmo Bio USA, AXS-1002424) in a 15 mL conical tube. The tube was centrifuged for 40 min at 4 °C (4700 x g). The layer corresponding to microbiota was extracted and washed with PBS for 3 times. The cells were directly processed for hydrogel encapsulation or stored in DNA/RNA shield (Zymo Research, R1100-50) at -80 °C for long term storage.

#### High-throughput single bacterial sequencing

Barcoded single cell bacterial sequencing libraries were constructed by modified SiC- protocol Seq leveraging Mission Bio Tapestri ([Lan et al., 2017](#ref-Lan2017)).

#### Cell encapsulation in hydrogel beads

Cell suspension (100 million per mL in PBS, 500 µL) was mixed with 500 µL of polyacrylamide precursor solution with 12% acrylamide(Thermo Scientific, AAJ62480AP), 1% N,N′-bis(acryloyl)cystamine (Sigma, A5912), 20 mM Tris (pH 8.0), 0.6% sodium persulfate (Sigma, 216232), and 20 mM NaCl. After adding 1mL of HFE 7500 with 2% surfactant (008-FluoroSurfactant, RanBiotechnologies), heterogenous droplets were generated by passing the oil/aqueous mixture through a syringe with 23.5 G blunt needle for 5 time. 20 µL of N,N,N′,N′- tetramethylethylenediamine (Sigma, 411019) was added into the emulsion and the emulsion was incubated at 70 °C for 30 min and at room temperature for 1 hour for gelation. The emulsion was centrifuged at 1000xG for 10 min and the oil layer was removed by a gel loading tip. To the hydrogel layer, 1 mL of 20% PFO (1H,1H,2H,2H-perfluoro-1-octanol, Sigma, 370533) in HFE 7500 and the mixture was vortexed and shaking for 10 min to break the emulsions. After centrifugation at 1000xG for 10 min, PFO were removed, and the hydrogel beads were then washed with PBS with 0.4% tween 20 for 3 times. The beads were then resuspended in 40% sucrose in PBS with 0.4% tween 20. A differential velocity centrifugation was performed to select the hydrogel beads within the size between 5 to 25 µm.

#### Cell lysis and DNA purification in hydrogel beads

100 µL of beads were treated in a solution of 1 mL TE buffer solution containing 2.5 mM EDTA (Teknova), 10mM NaCl (Sigma-Aldrich), 2U zymolyase, 5 U Lysostaphin, 50 U mutanolysin, and 20 mg Lysozyme at 37 °C overnight. The lysate mixture was then centrifuged at 3000 xG for 3 min, the supernatant removed, and 1 mL of TE buffer with 4U of Proteinase K, 1% triton X100 and 100 mM of NaCl was added to digest cellular proteins. The solution is incubated at 50 °C for 30 min. Following lysis, the beads was thoroughly washed to ensure complete removal of detergents and other chemicals which may inhibit the downstream reactions. The washes were performed in 10 mL volume with centrifugation magnitudes of 3000 x g between washes.

#### Tagmentsation reagents

25 µL Blocked ME Complement /5Phos/C*T* G*T*C* T*C*T* T*A*T* A*C*A*/3ddC/ (200 nM, IDT), 25 µL Tn5-Fwd-oligo GTACTCGCAGTAGTCAGATGTGTATAAGAGACAG (100 nM, IDT), and 25 µL Tn5-Rev-oligo TACCCTTCCAATTTAACCCTCCAAGATGTGTATAAGAGACAG (100 nM, IDT) and 25 µLTris buffer were mixed well in a PCR tube by pipetting. The mixture was incubated on a thermal cycler using the following program: 85°C for 2 min, cools to 20 °C with a ramping rate at 0.1 °C/s, 20 °C for 1 min, then hold at 4 °C with lid at 105°C. 100 µL of glycerol was then added into the annealed oligo. The unloaded Tn5 (1 mg/mL, expressed by QB3 MacroLab, Berkeley, CA.) was diluted at a 1:1 ratio in the Illumina dilution buffer (50% Glycerol, 100 mM NaCl, 0.1 mM EDTA, 1 mM DTT, and 0.1% NP40 in 50 mM Tris-HCl pH 7.5 buffer), followed by mixing at a 1:1 ratio with the pre-annealed adapter/glycerol mix. The mixture was incubated at room temperature for 30 min then stored at -20 °C.

#### Single cell DNA tagmentaion

The beads were resuspended in the density matching buffer (10 mM MgCl2, 1% NP40, and 17% Optiprep in 20 mM TAPS pH 7.0 buffer) to the final cell density of 3000 cells/µL. 25 µL of assembled Tn5 was mixed with 25 µL of tagmention buffer (10 mM MgCl2, 10 mM DTT in 20 mM TAPS pH 7.0 buffer). The MissionBio Tapestri DNA cartridge and a 0.2 mL PCR tube were mounted onto the Tapestri instrument. 50 µL of the beads in density matching buffer was loaded into the reservoir 2 (the reservoir for cell suspension), 50 µL of the Tn5 in tagmentation buffer was loaded into reservoir 1 (the reservoir for lysis buffer), and 200 µL of Encapsulation oil was loaded into reservoir 3. After applying the DNA gasket on top of the cartridge and closing the instrument lid, droplets were generated by running the Step1: Encapsulation program. The droplets were incubated at 37°C for 60 min and then 50°C for 30 min.

#### Barcoding PCR

Barcoding droplet PCR were performed according to the MissionBio Tapestri protocol with minor modification. 8 PCR tubes and DNA cartridge were mounted onto the Tapestri instrument. 200 µL and 500 µL of Electrode solution were loaded into reservoirs 4 and 5 respectively. After running the Priming program, 5 µL of reverse barcoding oligo was mixed with 295 µL MissionBio Barcoding Mix and loaded into reservoir 8 of the DNA cartridge. The droplets from previous step (~80 uL), 200 µL of barcoding beads, and 1.25 mL of Barcoding oil were loaded into reservoir 6, 7 and 9, respectively. After applying the DNA gasket on top of the cartridge and closing the lid, the droplets were merged with barcoding beads and PCR reagents by running the Cell Barcoding program on the Tapestri instrument. The droplets collected in the 8 PCR tubes were treated with UV for 8 min (Analytik Jena Blak-Ray XX-15L UV light source) and the bottom layer of oil in each tube were removed using a gel loading tip to leave up to 100 µL of droplets. The tubes were then thermo-cycled on a PCR instrument with the following program: 10 min at 72°C for 1 cycle, 3 min at 95°C for 1 cycle, (15 s at 95°C, 15 s for 55°C, and 2 min at 72°C) for 20 cycles, and 5 min at 72°C for 1 cycle.

#### Sequencing library preparation

The thermal cycled droplets in the 8 PCR tubes were carefully transferred into two 1.5 mL centrifuge tubes (4 PCR tube content in each). If there were merged droplets present, they were carefully removed using a 2 µL pipette. 20 µL PFO were added into each tube and mixed well by vortex. After centrifuging the top aqueous layers in each tube was transferred into new 1.5 mL tubes and water was added to bring the total volume to 400 µL. The barcoding product was purified using 0.7X Ampure XP beads (Beckman Coulter, A63882) and eluted into 60 µL H2O and stored at -20°C until next step. The concentrations of the barcoding product were measured with Qubit™ 1X dsDNA Assay Kits (ThermoFisher, Q33230).

The sequencing library were then prepared by attaching P5 and P7 sequences to the barcoding products using Nextera primers. The library PCR reactions were performed with 25 uL Kapa HiFi Master mix 2X, 5 uL Library P5 index primer (4 uM), 5 uL Library P7 index primer (4 uM), 10 uL purified barcoding products (normalized to 0.2 ng/uL), and 5 uL of nuclease free water. The PCR tubes were thermal cycled with the following program: 3 min at 95°C for 1 cycle, (20 s at 98°C, 20 s for 62°C, and 45 s at 72°C) for 10 cycles, and 2 min at 72°C for 1 cycle. The sequencing library was purified with 0.69X Ampure XP beads and eluted into 12 uL nuclease-free water. The library was quantified with Qubit™ 1X dsDNA Assay Kits and DNA HS chips on bioanalyzer or D5000 ScreenTape (Agilent, 5067- 5588) on Tapestation (Agilent, G2964AA). The libraries were pooled and paired-end sequenced by Novogene with a partial lane on Illumina NovaSeq 6000.

#### Single cell read files preparation

Sequencing data were processed using a custom python script (mb_barcode_and_trim.py) available on GitHub at <https://github.com/AbateLab/MissonBioTools>. For all reads, combinatorial cell barcodes were parsed from Read 1, using cutadapt (v2.4) and matched to a barcode whitelist. Barcode sequences within a Hamming distance of 1 from a whitelist barcode were corrected. Reads with valid barcodes were trimmed with cutadapt to remove 5′ and 3′ adapter sequences and demultiplexed into single-cell FASTQ files by barcode sequences using the script demuxbyname.sh from the BBMap package (v.38.57).

## Supplementary Results


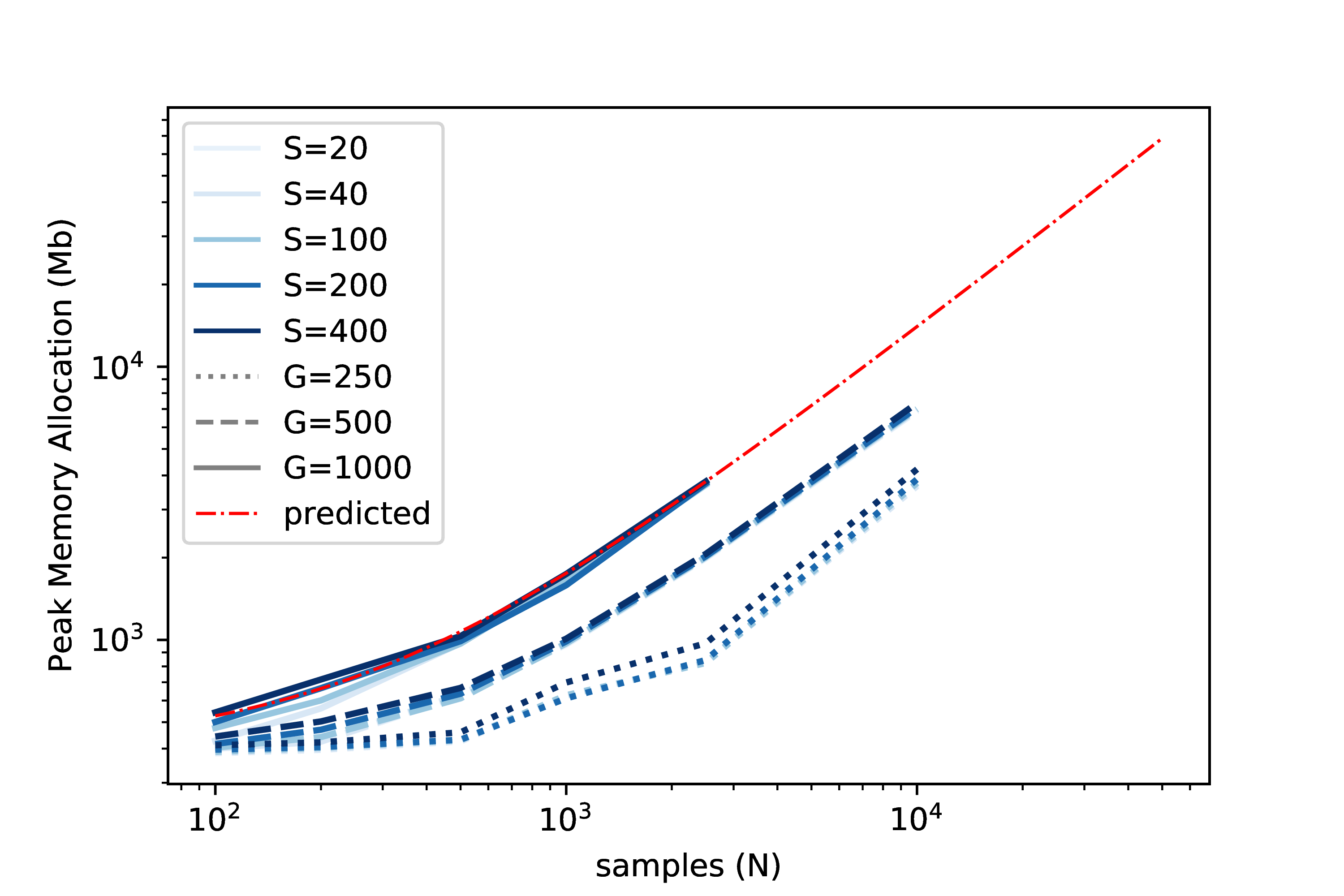


**Figure S2:** Maximum memory allocation across varying numbers of strains (S, line shade), SNPs (G, line style), and samples is plotted for StrainFacts models. Median of 9 replicate runs is shown. Maximum memory requirements are extrapolated to higher numbers of samples for a model with 1000 SNP sites (red line). An abridged version of this plot is included as Fig. 1.


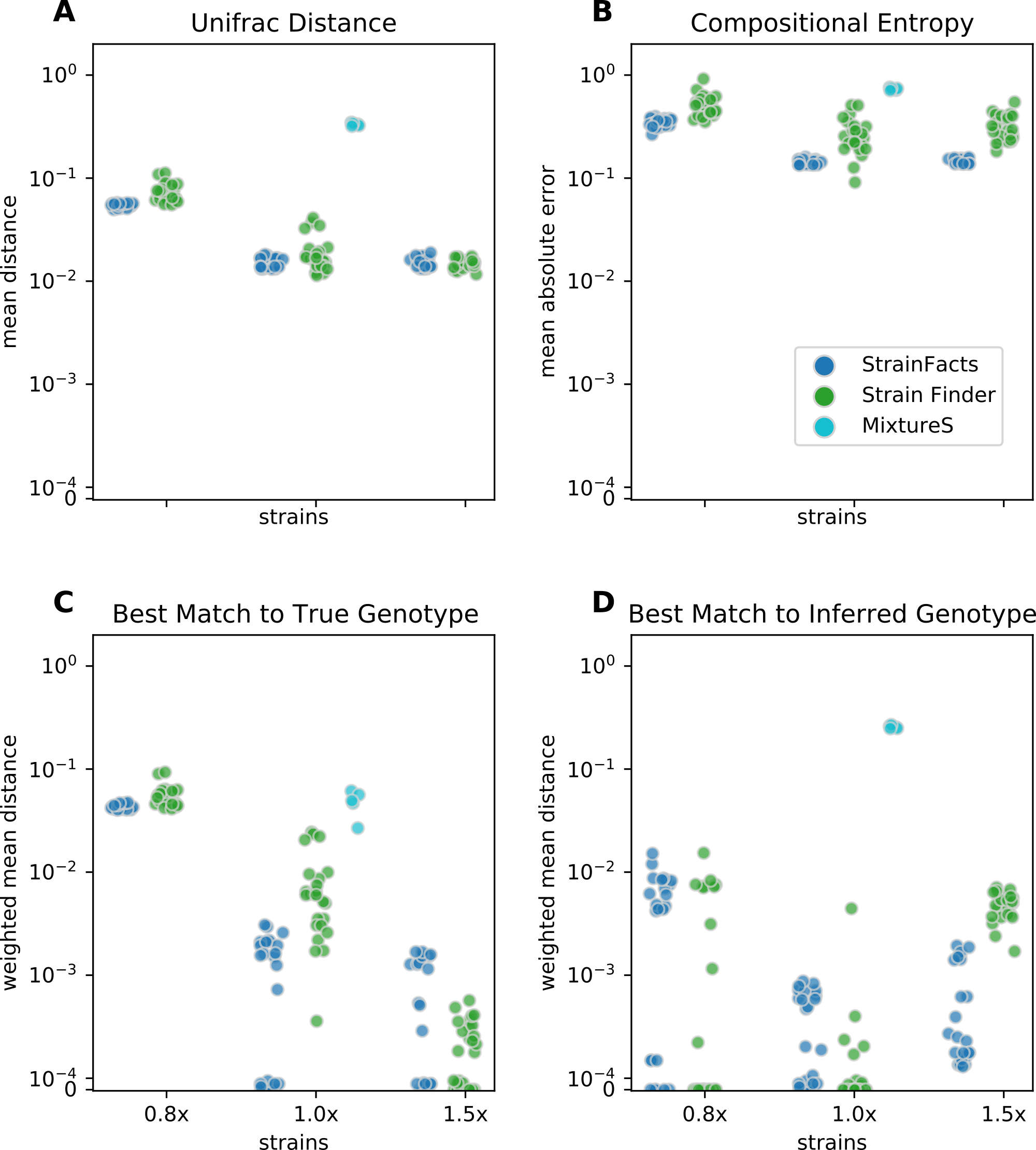


**Figure S3:** Extension of accuracy evaluation for StrainFacts and Strain Finder with additional results for MixtureS. Results are identical to panels A, C, D, and E in Fig. 2 (here panels **A**-**D**, respectively). Simulations are shown for five simulations with 250 SNP positions, 200 samples, and 40 strains. While StrainFacts and Strain Finder each have 32, 40, and 60 strains specified (the 0.8x, 1.0x, 1.5x parameterizations), MixtureS does not specify the number of strains a priori, and points are arbitrarily placed with the 1x parameterization. Similarly, MixtureS runs are deterministic; hence only one fit for each of the five simulations is shown.


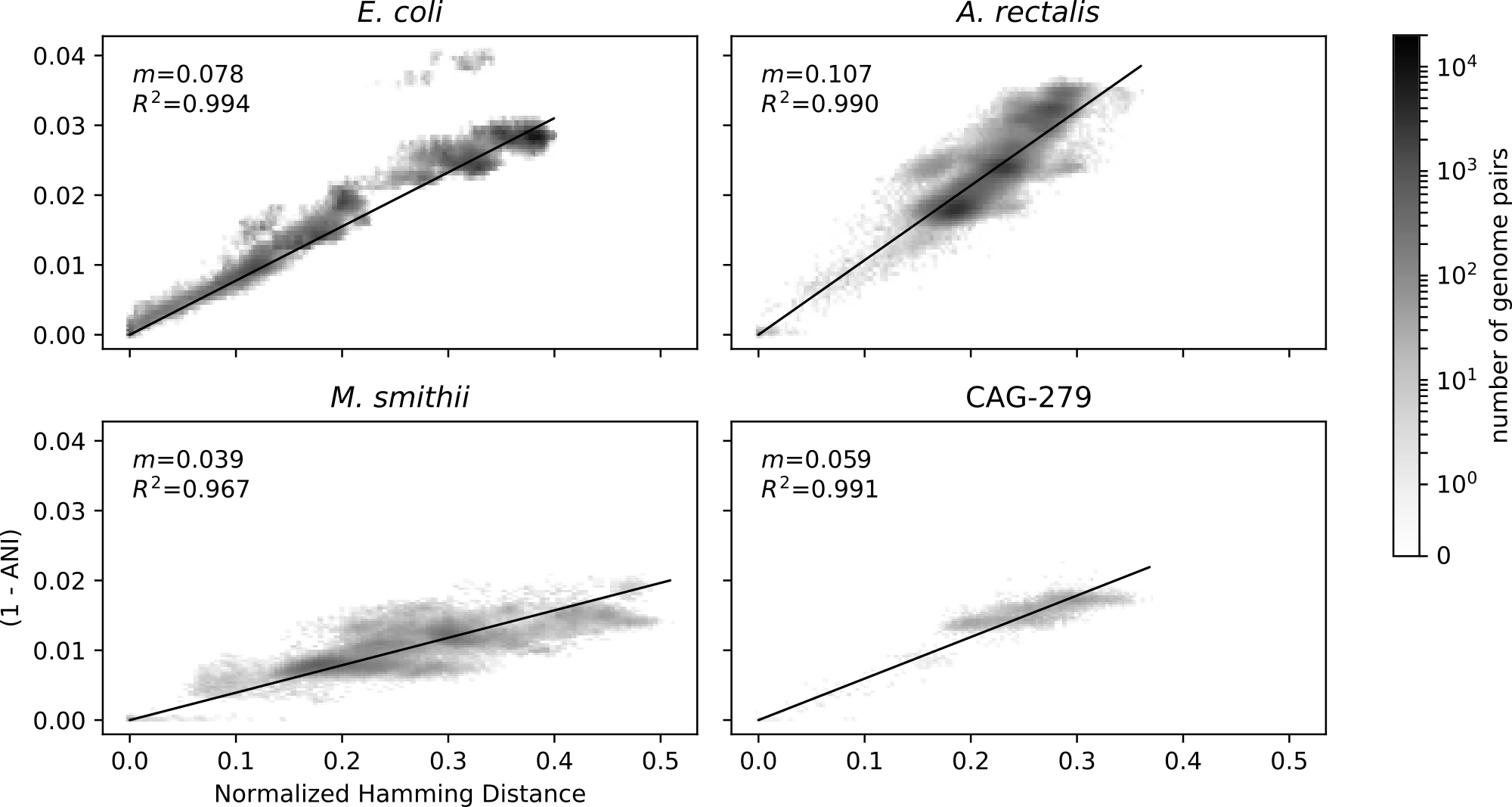


**Figure S4:** Empirical relationship between ANI and genotype distance among reference genomes in the GT-Pro database. Genotype distance is defined as the normalized Hamming distance at SNP sites considered by GT-Pro. All pairwise genome comparisons are plotted as a 2D histogram, with greater density indicated with darker colors. For each species, a linear regression calculated without an intercept term is shown (black line), and the constant of proportionality and uncentered R^2^ is also indicated.


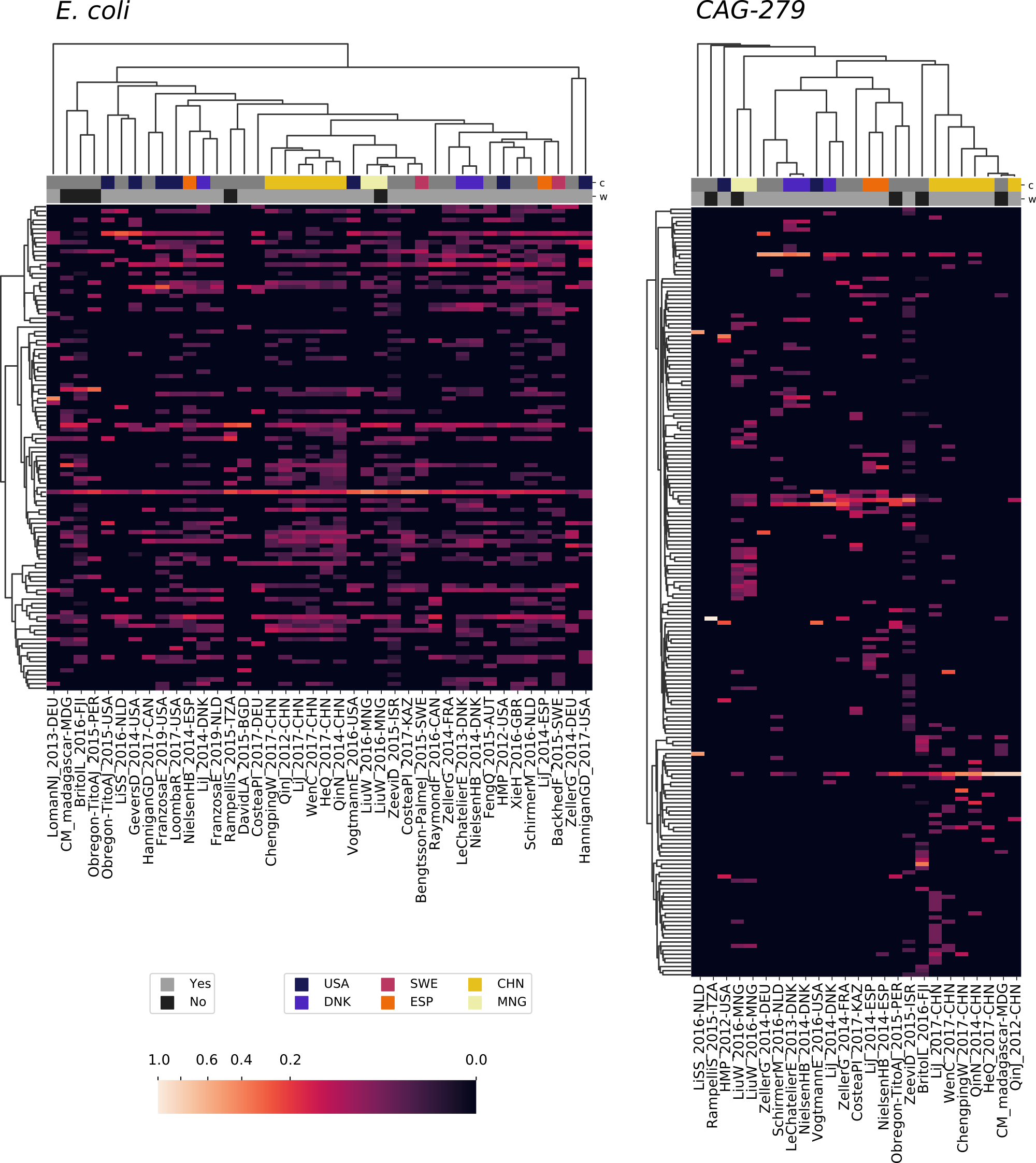


**Figure S5:** Patterns in strain dominance according to geography and lifestyle across thousands of publicly available metagenomes in dozens of independent studies for two additional members of the human gut microbiome. Visual elements are identical to Fig. 5: Columns represent collections of samples from individual studies and are further segmented by country and lifestyle (westernized or not). Rows represent strains inferred by StrainFacts. Cell colors reflect the fraction of samples in that study segment with that strain as the most abundant member. Study segments are omitted if they include fewer than 10 samples. Row ordering and the associated dendrogram reflect strain genotype distances, while the dendrogram for columns is based on their cosine similarity. Colors above the heatmap reflect the country in which samples were collected as well as whether samples were collected from individuals with a westernized lifestyle. Both a study identifier and the ISO 3166-ISO country-code are included in the column labels.

# References

Aitchison, J. (1986). *The statistical analysis of compositional data*. London; New York: Chapman and Hall.

Pedregosa, F., Varoquaux, G., Gramfort, A., Michel, V., Thirion, B., Grisel, O., et al. (2011). Scikit-learn: Machine learning in Python. *J. Mach. Learn. Res.* 12, 2825–2830. Available at: <http://www.jmlr.org/papers/v12/pedregosa11a.html>.

Neuwald, A. F., and Liu, J. S. (2004). Gapped alignment of protein sequence motifs through Monte Carlo optimization of a hidden Markov model. *BMC Bioinformatics* 5, 157. doi:[10.1186/1471-2105-5-157](https://doi.org/10.1186/1471-2105-5-157).

Hevia, A., Delgado, S., Margolles, A., and Sánchez, B. (2015). Application of density gradient for the isolation of the fecal microbial stool component and the potential use thereof. *Sci Rep* 5, 16807. doi:[10.1038/srep16807](https://doi.org/10.1038/srep16807).

Lan, F., Demaree, B., Ahmed, N., and Abate, A. (2017). SiC-Seq: Single-cell genome sequencing at ultra high-throughput with microfluidic droplet barcoding. *Nat. Biotechnol.* 35, 640. doi:[10.1038/nbt.3880](https://doi.org/10.1038/nbt.3880).
